# Healthcare center-based cell therapy laboratories supporting off-site manufactured cell therapies: The experiences of a single academic cell therapy laboratory

**DOI:** 10.1111/trf.17703

**Published:** 2024-01-03

**Authors:** Anh Dinh, David F. Stroncek

**Affiliations:** Center for Cellular Engineering, Department of Transfusion Medicine, NIH Clinical Center, Bethesda, Maryland, USA

**Keywords:** cell therapy, logistics, off-site manufacturing

## Abstract

**Background:**

Healthcare center-based cell therapy laboratories (HC CTLs) evolved from solely processing hematopoietic stem cells for transplantation to manufacturing various advanced cellular therapies. With increasing interest in cellular therapy applications, off-site manufactured products are becoming more common. HC CTLs play a critical role in supporting these products by shipping out cellular starting material (CSM) for further manufacturing and/or receiving, storing, and distributing final products. The experiences and challenges encountered by a single academic HC CTL in supporting these products are presented.

**Methods:**

All off-site manufacturing protocols supported before 2023 were reviewed. Collected data included protocol characteristics (treatment indication, product type), process logistics (shipping, receiving, storage, thawing, distribution, documentation), and product handling volumes (CSM shipping and final product infusions).

**Results:**

Between 2012 and 2022, 15 off-site manufactured cellular therapy early-phase, single- and multicenter clinical trials were supported. Trials were sponsored by academic/research and commercial entities. The number of protocols supported annually increased each year, with few ending. Products included cancer immunotherapies and gene therapies. Autologous CSM was collected and shipped, while autologous and allogeneic final products were received, stored, thawed, and distributed. Process differences among protocols included CSM shipping conditions, laboratory analyses, final product thaw conditions and procedures, number of treatments, and documentation.

**Discussion:**

HC CTLs must contend with several challenges in supporting off-site manufacturing protocols. As demand for cellular therapies increases, stakeholders should collaborate from the early phases of clinical trials to streamline processes and standardize procedures to increase value, improve safety, and reduce the burden on HC CTLs.

## 1 | INTRODUCTION

Healthcare center-based cell therapy laboratories (HC CTLs) have evolved from solely processing hematopoietic stem cells for transplantation to manufacturing various advanced cell therapies, including mesenchymal stromal cells, virus-specific T-cells (VSTs), gene-corrected hematopoietic stem cells, induced pluripotent stem cell-derived regenerative therapies, and cellular cancer immunotherapies. While these therapies were initially produced primarily at academic medical centers for on-site clinical trials, a burgeoning interest in cell therapy applications in the past decade has led to a landscape shift and surge in the therapies being developed. As of 2022, there were more than 2700 active cell therapies in the immuno-oncology pipeline and nearly 1800 active cell therapy trials registered on Clinicaltrails.gov.^1^ With growing demand, cell therapies manufactured at one center are often shipped to other treatment centers as a part of single- or multicenter clinical trials. Beyond academic clinical settings, commercial groups are also actively involved in developing these advanced cell therapies. As of this writing, the U.S. Food and Drug Administration (FDA) has approved six commercial chimeric antigen receptor (CAR) T-cell products.^2^

**FIGURE 1:**
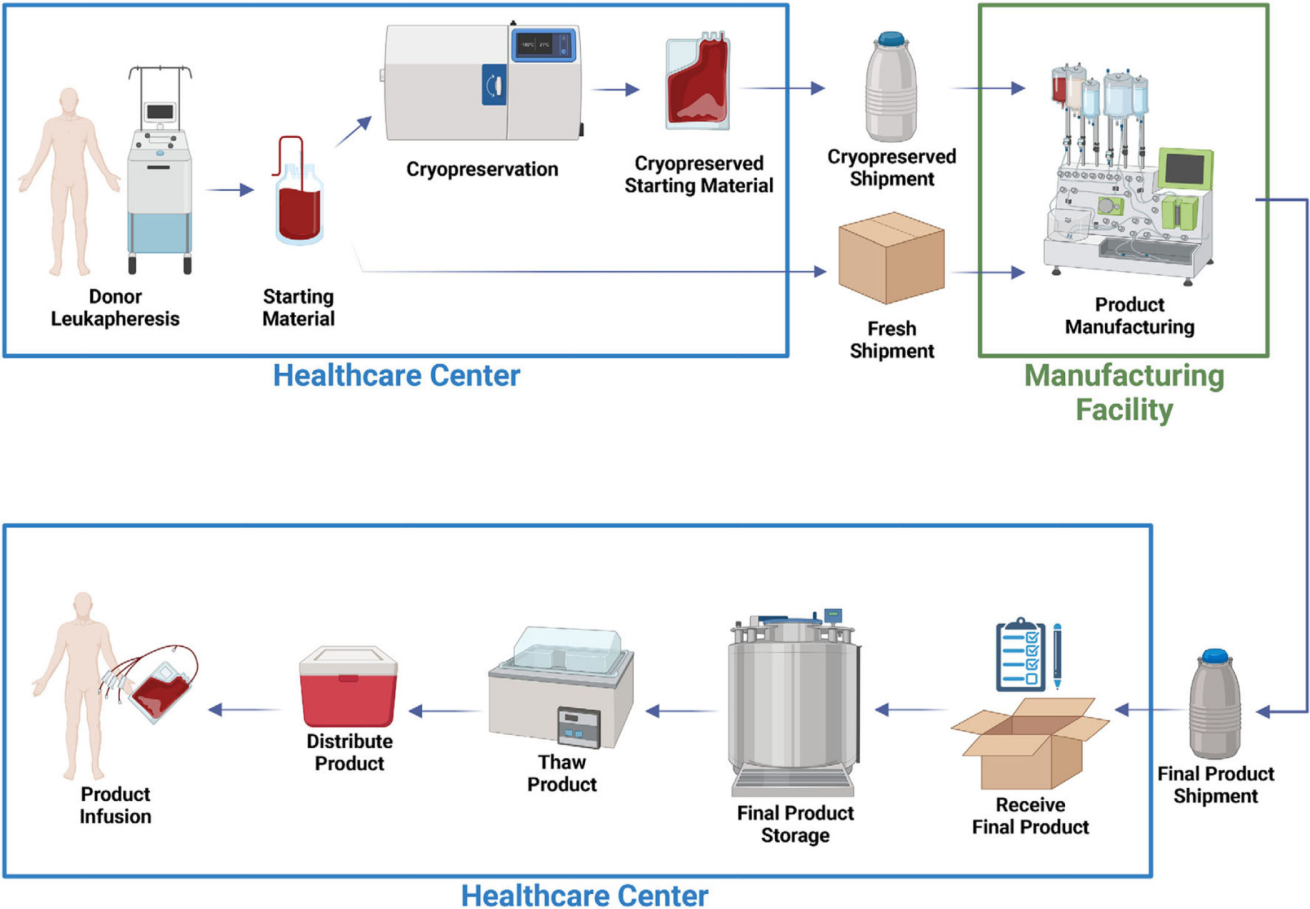
End-to-end schematic for off-site manufactured autologous cell therapy products (Created with Biorender).

The widespread use of cell therapies as investigational or licensed treatments has required HC CTLs to collaborate with off-site centralized manufacturing facilities to transport, store, and distribute cellular therapies (Figure 1). As a starting point for autologous therapies, the HC CTL may need to coordinate with its donor apheresis or blood collection team to collect cellular starting material (CSM). The HC CTL may also need to perform laboratory analyses on the CSM, such as complete blood counts and flow cytometry, before shipping it fresh or cryopreserved to the off-site manufacturing facility. Following manufacturing, the off-site facility returns the final product to the HC CTL for receipt, storage, and distribution to the patient care unit for infusion. Some cellular therapies, such as allogeneic universal recipient products, may only require the HC CTL to receive, store, and distribute the final cell therapy product without prior upstream involvement.

To support off-site manufactured cellular therapies, HC CTLs must dedicate substantial resources and overcome several challenges. Beyond generic considerations to support these processes, various manufacturing groups may have distinct procedures and specifications for otherwise similar processes. The inevitable lack of standardization requires the HC CTL to constantly develop new systems, allocate sufficient support staff, and maintain competency, which can be especially difficult for products handled infrequently.^3^

The Center for Cellular Engineering (CCE) within the Department of Transfusion Medicine at the National Institutes of Health (NIH) has supported early-phase clinical trials involving cellular therapies, including the processing of standard transplant products as well as the manufacturing of investigational new drug (IND) products. Since 2012, the CCE has also supported protocols involving cellular therapy products manufactured off-site.^4^ We present an overview of the nature of off-site centrally manufactured cellular therapy products, their growth, and the challenges encountered by the CCE in handling these products.

## 2 | METHODS

All off-site manufacturing protocols supported by the CCE before 2023 were retrospectively reviewed. Protocols and associated documents (product manuals, forms) were reviewed to collect data on protocol characteristics such as the treatment indication, product type, and process logistics, including shipping, receiving, storage, thawing, and distribution. Product handling volumes (CSM shipping and final product infusions) were obtained from the laboratory information system StemLab version 3.6.2, StemSoft (Vancouver, British Columbia, Canada). Data collection, statistical analysis, and graphic presentation were performed using computer software (Excel, Microsoft (Redmond, WA), GraphPad Prism version 9.5.1, GraphPad Software (San Diego, California)), and SankeyMATIC (http://sankeymatic.com).

## 3 | RESULTS

### 3.1 | Protocol characteristics

**FIGURE 2:**
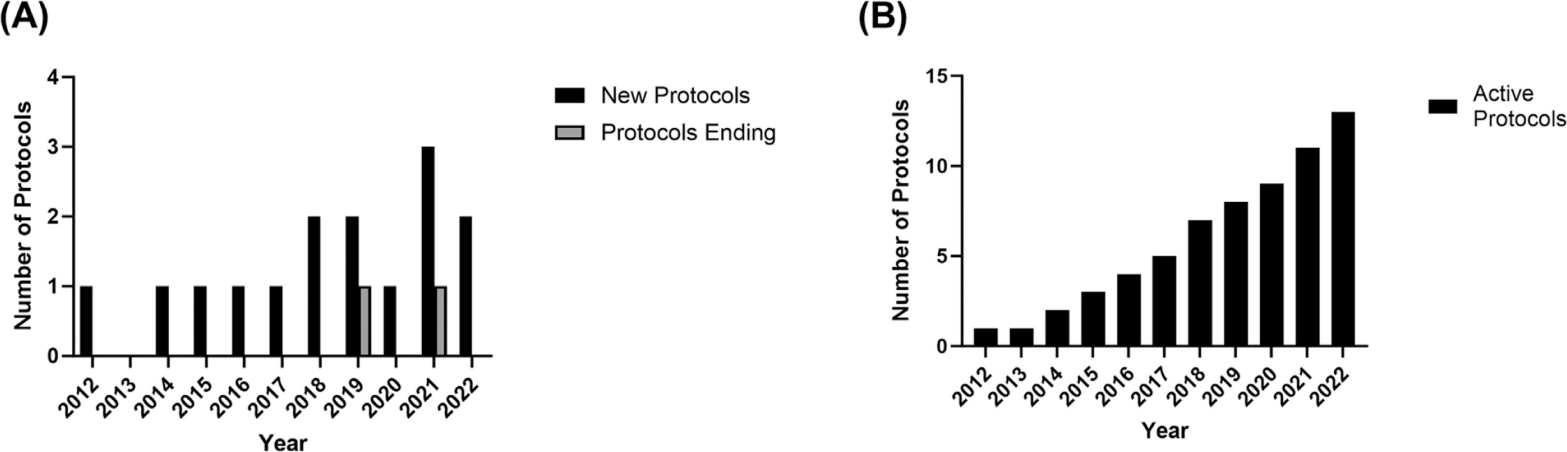
Protocols handled each year. (A) Number of new protocols and protocols ending. (B) Number of active protocols supported per year.

The CCE supported 15 off-site manufacturing protocols between 2012 and 2022, with a median of 1 (range 0–3) new protocol a year and only two protocols ending during this timeframe (Figure 2A). The number of active protocols increased annually, starting with one in 2012 and reaching 13 in 2022 (Figure 2B).

All protocols were early phase I and II clinical trials sponsored by academic/research institutions and commercial entities, including single- and multicenter studies (Table 1). Commercial laboratories comprised the majority of the manufacturing facilities, compared to the academic/research centers. Treatment indications included hematologic disorders/malignancies and solid tumors.

### 3.2 | Types of off-site-manufactured cell therapy products

**FIGURE 3:**
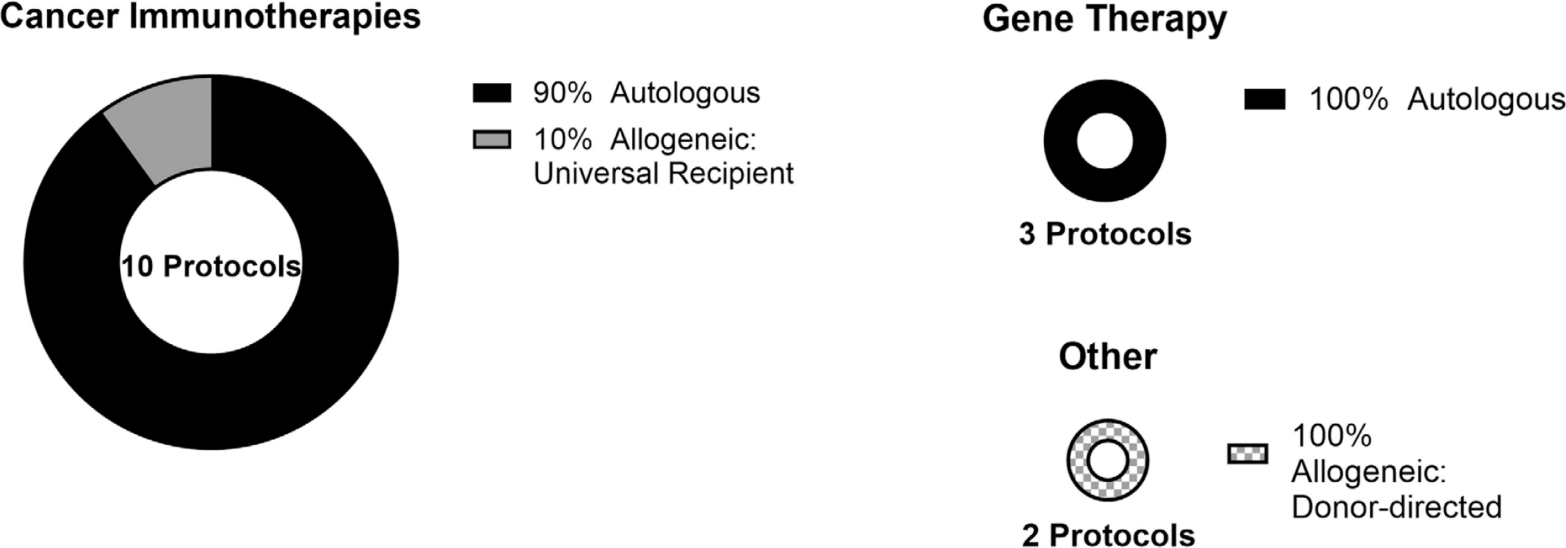
Types of cell and gene therapies.

Various cell therapy products were manufactured off-site, including cancer immunotherapies and gene-corrected hematopoietic stem cells (ex., sickle cell disease) (Figure 3). The majority of these therapies were autologous. Allogeneic cell therapies included both donor-directed and universal recipient “off-the-shelf” products. Donor-directed products, such as gene-edited peripheral blood stem cells (PBSCs) and ex-vivo expanded cord blood, required at least partial human leukocyte antigen (HLA) matching and were used for transplantation for a specific recipient. Upcoming protocols involve handling VSTs prepared from healthy donor cells with some degree of shared expression of HLA antigens between donor and recipient. Universal products, such as genetically-engineered Natural Killer cells, are genetically modified for any recipient to use. Our center did not handle FDA-licensed cellular therapies.

### 3.3 | Differences in the process of handling cellular starting material (CSM)

Handling cell therapy products manufactured off-site is complex, compounded by the variety of products, shipping conditions, and testing requirements. All autologous CSM are collected via apheresis by our internal apheresis department. Peripheral blood mononuclear cells (PBMCs) or mobilized PBSCs are used to manufacture engineered T-cells (CAR T-cells) and gene-corrected hematopoietic stem cells, respectively. Our center did not collect allogeneic CSM.

**FIGURE 4:**
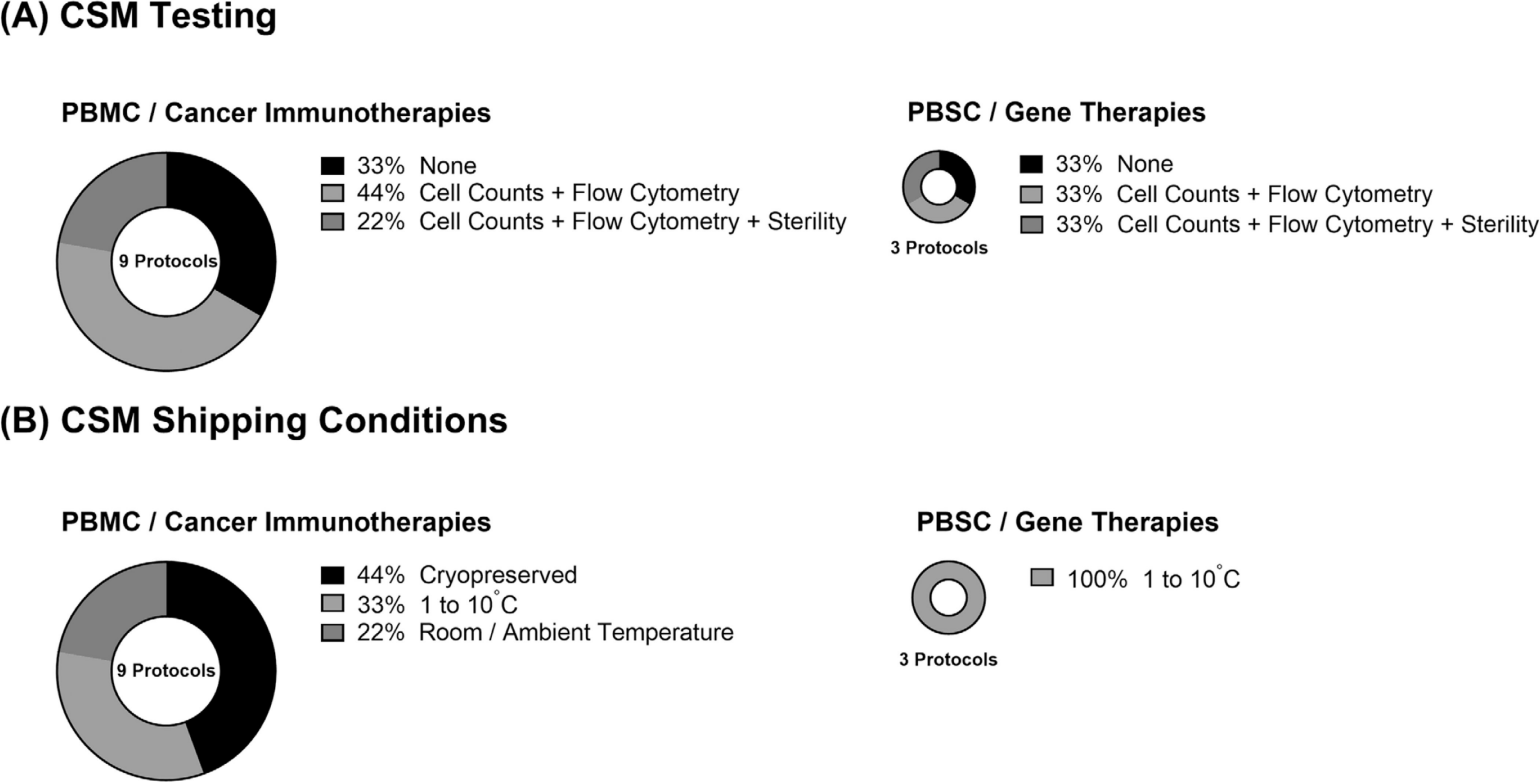
Cellular starting material (CSM) requirements for shipping to external Manufacturing Facilities. (A) CSM testing required by protocol, by CSM type and product type. It is our center-specific practice to routinely perform cell counts and sterility on apheresis starting material. (B) Shipping conditions required by protocol, by CSM type and product type. PBMC-Peripheral blood mononuclear cells. PBSC-Peripheral blood stem cells.

For many of the 12 protocols using autologous CSM, CCE was responsible for testing the material before shipping. The nature of this testing varied depending on the protocol (Figure 4A). Results were sent electronically to the manufacturing laboratory before or after the shipment of CSM, or as hard copies with the shipment.

The shipping conditions of CSM also varied, with some protocols requiring cryopreservation before shipment, and others specifying shipping between 1 to 10°C or at room/ambient temperature (see Figure 4B). To accommodate these specifications, each manufacturing site sent an appropriate shipping container to CCE, where it was received and held until the shipment.

### 3.4 | Differences in the process of handling the final cellular therapy product

**FIGURE 5:**
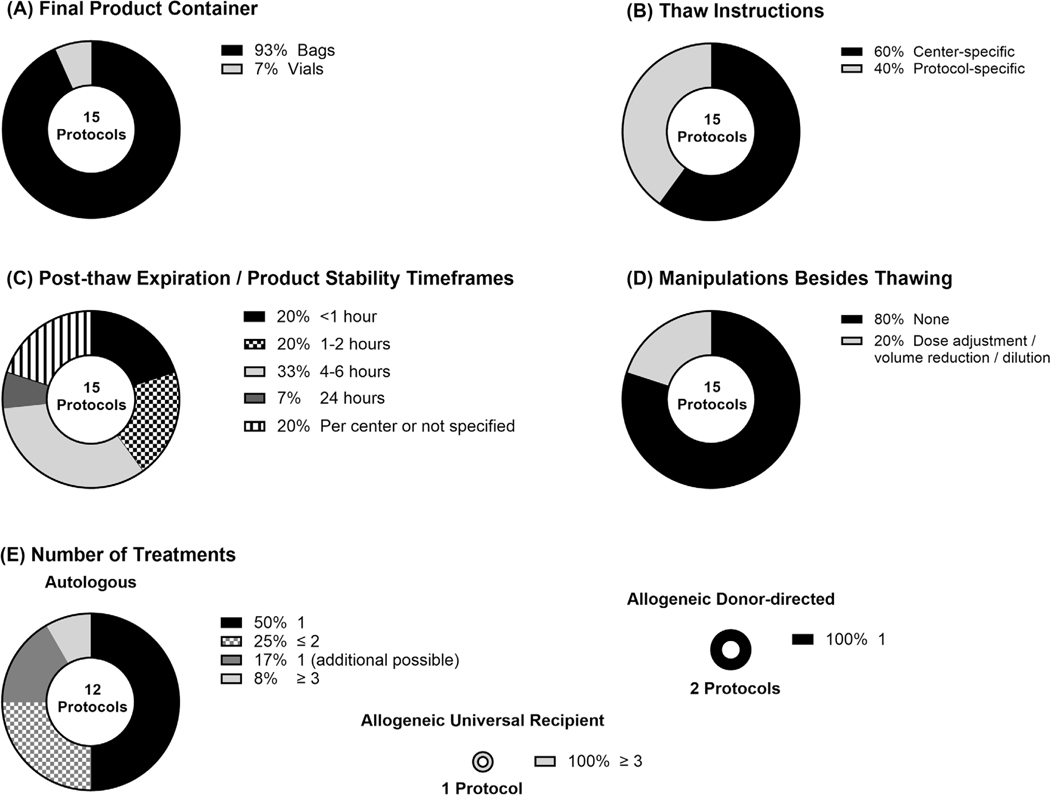
Final products received from external manufacturing facilities. (A) Final product container. (B) Thaw instructions. (C) Post-thaw expiration and/or product stability timeframes. (D) Final product manipulations besides thawing. (E) Number of possible treatments by cell source: autologous, allogeneic donor-directed, allogeneic universal recipient.

Similar to the first leg of the process involving the handling of CSM, the second leg involving the receipt, storage, and distribution of the final product also had protocol-dependent variabilities. All final products received from the off-site manufacturing facilities were cryopreserved (although some upcoming protocols involve receiving and issuing cell therapies stored at room temperature or 1 to 10°C). The containers used for the final product also varied, which for most protocols were bags (Figure 5A). However, products were packaged in vials in one protocol involving allogeneic universal recipient cells (and some upcoming protocols involving VSTs).

The thaw instructions for cryopreserved final products also differed between protocols (Figure 5B), with some permitting the use of center-specific standard methods (9 of 15, 60%), and others with protocol-specific instructions (6 of 15, 40%). Although all cryopreserved cell therapy products were thawed in our laboratory, some protocols permitted thaw and infusion at the patient’s bedside.

Additionally, the duration of post-thaw product stability was variable, with some products having specified expiration times and others having product stability data with ideal post-thaw infusion timeframes. The definitions of “post-thaw” also differed, such as upon removing the frozen product from liquid nitrogen storage for thawing or removing a thawed product from the water bath. Protocol-specified expiration and/or stability timeframes included durations as short as 40 min and as long as 24 h (Figure 5C). In addition to hard expiration or product stability durations, some protocols also specified ideal timeframes to target between thawing and completion of infusion, ranging from 5 min to 1–2 h.

Post-thaw, most cell therapy products could be administered via simple spiking of the container with an administration set (*n* = 12) (Figure 5D). However, for specific protocols that required dose adjustments (*n* = 3), HC CTL processing staff reduced the volume of the final product bag before issuing. For one protocol, post-thaw products had to be diluted with a company-provided diluent before being issued. None of the protocols required transferring the product to a new container for issuing, although a few upcoming protocols will require such transfer.

While patients were typically treated only once with autologous cell therapies, some protocols allowed multiple treatments (Figure 5E). One protocol involving an allogeneic product allowed for treatments every one to two weeks until disease progression (one patient received 12 infusions in one year). Upcoming protocols involving allogeneic products also allow multiple treatments.

### 3.5 | Documentation

Documentation, a critical aspect of cellular therapy product handling, differed between protocols. Sponsors provided their own forms to capture information at various stages of the process. CSM collection and shipment forms typically included information about the subject (identifiers, height, weight) and apheresis product (collection date/time, volume, cell counts, cryoprotectant, etc.). Final product forms captured information related to the product’s receipt, thaw, and infusion. They also included product details, such as lot numbers, expiration dates, and storage conditions. Internal chain-of-custody and accountability logs for both legs of the overall process ensured product integrity and identity maintenance throughout.

Protocols (*n* = 15) had a median of 4 forms (range 2–8) for which staff were responsible for filling out information. These did not include documents requiring review or containing specific instructions for handling. While the information captured by forms in aggregate was similar between protocols, no one individual form between sponsors captured the exact same information. As an example, for two of these protocols, “A” and “B,” while Protocol A’s “product accountability log” captured thaw start and stop times, Protocol B’s similarly named version of this form did not capture this information. Instead, the same thaw time information was captured on Protocol B’s “product thaw worksheet.” Similarly, documentation of deviations or issues during the handling process may occur in forms or through email notification. Some protocols also required that data be entered into a web-based form or database, which at our center, was handled by research coordinators. Finally, general product label information, such as the selected unique identifiers (patient name, date of birth, subject ID, etc.), also varied.

### 3.6 | Frequency of handling cellular therapy products and staffing support

**FIGURE 6:**
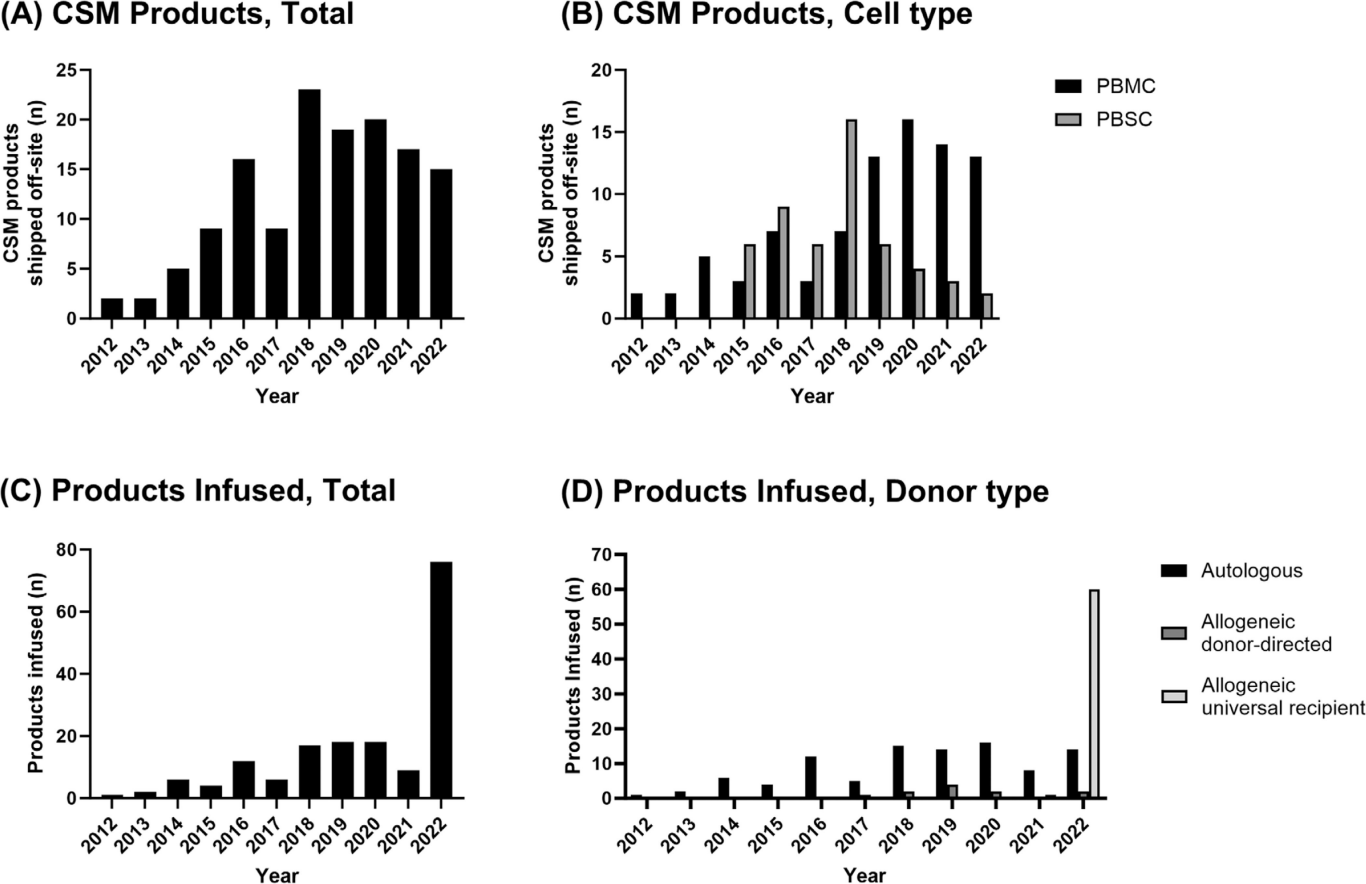
Number of products handled each year: CSM received and processed, and final products infused. (A) CSM products received and processed: total number each year and (B) number of PBMC and PBSC each year. (C) Total number of final products infused each year and (D) number infused by donor type. PBMC-Peripheral blood mononuclear cells. PBSC-Peripheral blood stem cells. CSM-Cellular starting material.

Over time, the number of cell therapy products handled by our center increased (Figure 6). In 2012, our center handled only two autologous CSM collections and one product infusion. By 2022, we handled 15 autologous CSM collections and 76 product infusions, primarily consisting of allogeneic products administered to patients every one to two weeks until disease progression. For active protocols, the median number of times our HC CTL handled a product each year was three (range 0–60).

The increased demand in 2022 impacted our operations. Our processing staff, who primarily handle in-house standard-of-care transplant products (hematopoietic stem cell products for transplant and donor lymphocyte infusions), also handle the off-site manufactured products. With the increased demand, processing staff who typically support our in-house IND products (CAR-T, T-cell receptor-based therapy, etc.) were also required to support off-site manufactured products (46 of 72 product infusions, 61%).

## 4 | DISCUSSION

**FIGURE 7:**
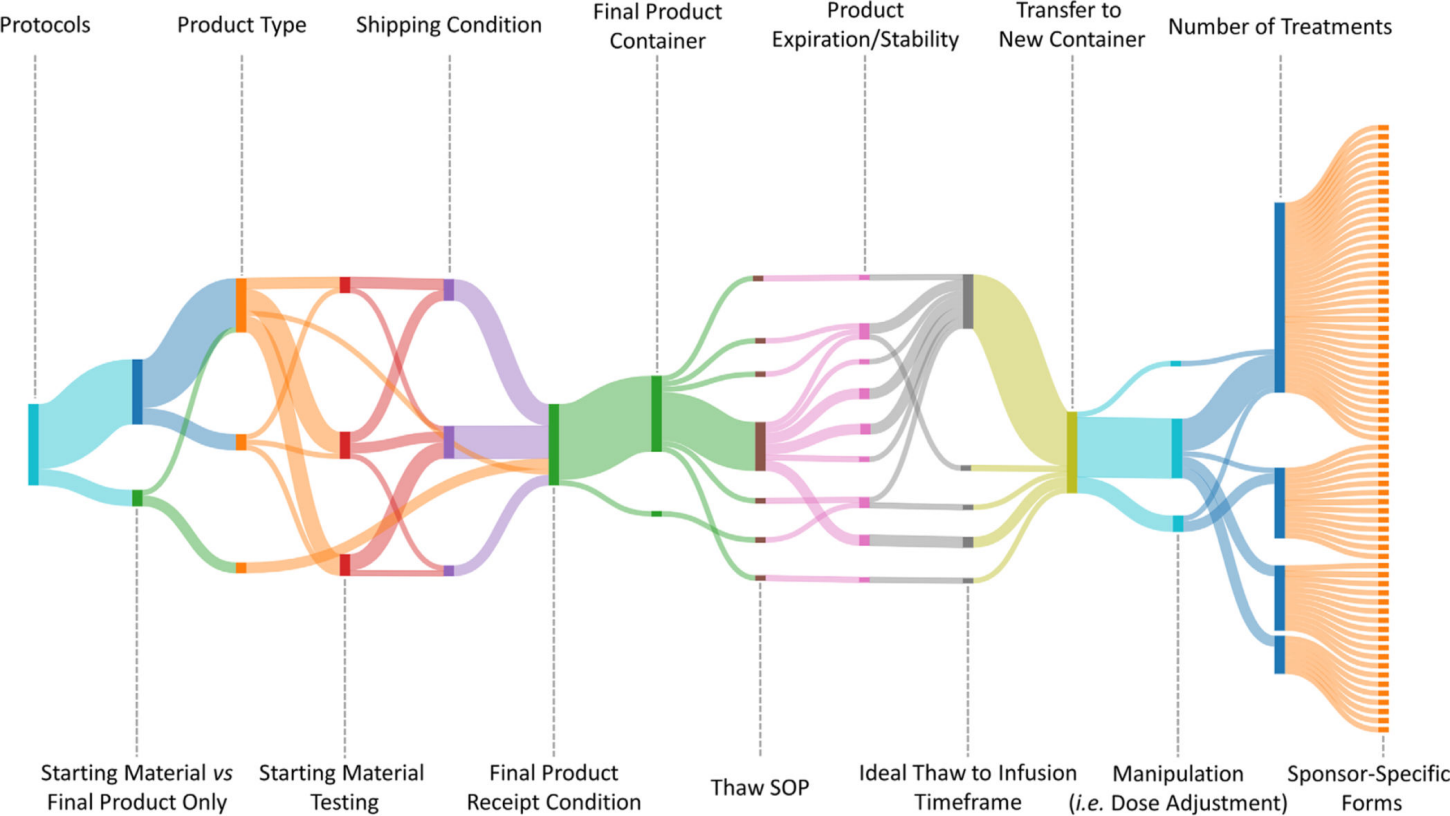
Overall summary of the variability that permeates the product handling process for off-site manufacturing protocols (15). Each node represents a category or step along the process and each branch represents a variation or difference in method (Created with SankeyMATIC). SOP-Standard operating procedure.

This report details the experience and challenges encountered by a single center in supporting early-phase clinical trials (Phase I and II) for cell therapies. The primary root of these challenges appears to be the variation permeating the entire product handling process, ranging from CSM testing and shipping conditions to thawing instructions and post-thaw product expiration times (Figure 7). Participating centers face the arduous task of navigating often ambiguous, varied, and overlapping expectations from off-site sponsors and manufacturers. These challenges are particularly apparent when handling products for any given protocol occurs only a few times per year, which was common at our center: a median of three products was handled for each protocol yearly and sometimes was not handled at all. Infrequent handling becomes a barrier to the maintenance of staff competency, and staff must reacquaint themselves with the process as products are received. This difficulty is further compounded by an increasing number of protocols supported annually, outpacing the resources and staffing dedicated to supporting these protocols. The combination of infrequent handling and an exponential increase in protocols raises concerns regarding process sustainability, product quality, and patient safety. Our challenges are similar to those described by other centers that handle commercial cell therapy products.^5,6^ In this report, we seek to provide a more detailed characterization of the process and product variability encountered for early-phase clinical trials to help other centers anticipate what this support entails and to optimize any stakeholder collaborations to streamline and standardize processes.

We have several strategies to mitigate the impact of these protocol differences. CCE has a service coordination team with subject matter expertise in navigating and onboarding off-site manufacturing protocols. This team serves as the liaison between study teams, external sponsors, and other CCE staff. They also schedule and communicate with external parties regarding all product handling-related events, such as starting material receipt, shipping, final product receipt, and distribution. The technologists supporting these products are also subject matter experts in handling off-site manufactured products. CCE also dedicates time to preparing for new protocols, which may be significant depending on the protocol complexity or the level of experience of sponsors and study teams. Protocol materials are reviewed, and sponsors are requested to complete a questionnaire about the support that CCE will be providing. The questionnaire contains information regarding scheduling, processing, testing, shipping, storage, thaw, distribution, quality assurance, information technology, forms, and labels. This document also provides center-specific practices and preferences to the sponsor, such as scheduling practices (product handling to occur on specific days) to align with our internal protocols and products. Other centers have reported similar strategies to prepare for supporting commercial products, which include processes for reviewing sponsor requirements, resource planning, training, and SOP development.^5,6^

As a part of the onboarding process, email communications and meetings are requested to clarify logistics or receive training. Sponsors may provide training in various forms (in-person, virtual meetings, videos, slides, etc.), and if necessary, we request more specific detailed training from the sponsor. Even after a protocol is active, we may request refresher training from a sponsor if a significant time has elapsed between handling products. For all protocols, we also participate in mock or dry runs of the process before handling clinical products to identify potential issues and for quality assurance. These runs are most effective when they closely mirror the future clinical product handling process. Once protocols are active, if a significant amount of time has elapsed since handling a product, re-training sessions are requested from the sponsor.

Policies and procedures are of critical importance in the preparation process. For each protocol, our center writes a document that integrates center-specific and sponsor-provided standard operating procedures (SOPs). These protocol-specific instructions allow our staff to handle a variety of protocols and products more readily. We typically advocate for using center-specific practices and procedures to reduce variability, which may, in our experience, work well for CSM collection, processing, and handling, but its feasibility depends on the flexibility granted by the sponsor’s IND application. With regard to handling final products, which have specific stability and expiration requirements, the ability to use center-specific approaches may be more limited. Additionally, when we first began supporting these protocols, they tended to be single-center protocols in which sponsors were more receptive to center-specific procedures because we were the only participating center. Over time, as multicenter protocols increased, sponsors sought to standardize processes across sites. Their standardization comes at the expense of individual participating centers, which are forced to invest time and resources to adapt. Not all centers will have the means to meet such demands. As such, standardization of logistical processes shared across multiple protocols and sponsors to the extent that is possible appears to be the best solution.

Beyond individual center-specific efforts, different professional organizations are trying to address the lack of standardization. For commercial cell therapies, the American Society for Transplantation and Cellular Therapy (ASTCT) convened an 80/20 Taskforce to examine the challenges and identify solutions for enhancing the efficiency of clinical center certification and maintenance of operations.^7^ Through discussions with clinicians, regulators, manufacturers, and professional societies, the taskforce concluded that 80% of manufacturers’ requirements for onboarding and ongoing operations of commercially available products could be standardized and streamlined. The taskforce also noted that many logistical nuances between different commercial therapies stem from what was practiced in pivotal registration investigational trials and become locked in at the time of FDA approval of the commercial biologic license application. This observation, along with our experience with early-phase trials, underscores the importance of stakeholders working to standardize processes from the outset by starting with early-phase trials to prevent downstream consequences for products that are ultimately commercialized.

Additional efforts have been made to address process differences, such as the Association for the Advancement of Blood and Biotherapies (AABB) CSM Forum, which focuses on standards development and industry and regulatory perspectives to prepare cell collection organizations for CSM qualification, which is increasingly sought after. Additionally, the AABB CSM Qualification program aims to accelerate cellular material supplier qualification for biotherapies companies that rely on AABB-accredited facilities to support advanced therapies.^8^

For future directions, one idea would be to learn from what has already been established. Several decades ago, the tremendous efforts of the National Marrow Donor Program (NMDP) established the first national registry of unrelated donors for hematopoietic cell transplantation.^9,10^ Up to this point, unrelated donor transplants were hindered by the immense logistical challenges posed by multiple concurrent smaller registries, each with its own policies and procedures for donor selection, graft procurement, and transport. In the years since, NMDP/Be The Match has overseen the development of standards, policies, and coordination of logistics in this space. A subsidiary, Be The Match Biotherapies, is also supporting the logistics for several cell therapy trials. Having a similar entity to more globally oversee the logistics of off-site manufactured products may be very fruitful, increasing efficiency for the overall process, and alleviating the burden for individual centers, who may not otherwise be able to dedicate the necessary resources.

As centers encounter demands to support these products, there may also be a request for so-called “legacy” services to take on new roles to better enable internalization. The logistics involved in handling products manufactured off-site are similar to the functions of established, existing hospital services such as the blood bank or pharmacy. For instance, handling products where a center is involved in collecting CSM is similar to the practices followed by blood collection centers. Similarly, the logistics of final products for specific intended recipients are comparable to blood component receipt, storage, and issuance. In contrast, the logistics involved in receiving, storing, and distributing universal recipient products are similar to the functions of a pharmacy. As the prevalence of off-site manufactured cellular therapies continues to increase, centers may discover new roles in which blood banks and pharmacies become involved. Standardizing these logistics would further facilitate such a transition.

Cell therapy products hold great promise in treating various diseases, and their use is expected to increase. HC CTLs play a critical role in supporting off-site centralized manufacturing of these products, but the logistics involved are complex and require significant resources. To reasonably accommodate the deluge of therapies, stakeholders must collaborate from the early phases of clinical trials to streamline processes, standardize procedures, increase value, and improve safety while reducing the burden on HC CTLs.

## Figures and Tables

**TABLE 1:** Characteristics of protocols.

Total (*n*)	15
Single-versus multicenter Single-center, *n* (%)	4 (27%)
Multicenter, *n* (%)	11 (73%)
Phase I, *n* (%)	6 (40%)
II, *n* (%)	2 (13%)
I/II, *n* (%)	7 (47%)
Sponsor Academic/Research, *n* (%)	6 (40%)
Commercial, *n* (%)	9 (60%)
Manufacturing Facility Academic/Research, *n* (%)	4 (27%)
Commercial, *n* (%)	11 (73%)
Treatment Indications Hematologic disorders/malignancies, *n* (%)	6 (40%)
Solid tumors, *n* (%)	9 (60%)

## Abbreviations:

AABB: Association for the Advancement of Blood and Biotherapies
ASTCT: American Society for Transplantation and Cellular Therapy
CAR: chimeric antigen receptor
CCE: Center for Cellular Engineering
CSM: cellular starting material
FDA: U.S. Food and Drug Administration
HC CTL: healthcare center-based cell therapy laboratories
HLA: human leukocyte antigen
IND: investigational new drug
NIH: National Institutes of Health
NMDP: National Marrow Donor Program
PBMC: peripheral blood mononuclear cells
PBSC: peripheral blood stem cells
VST: virus-specific T-cells
